# Prediction of transcript isoforms in 19 chicken tissues by Oxford Nanopore long-read sequencing

**DOI:** 10.3389/fgene.2022.997460

**Published:** 2022-10-03

**Authors:** Dailu Guan, Michelle M. Halstead, Alma D. Islas-Trejo, Daniel E. Goszczynski, Hans H. Cheng, Pablo J. Ross, Huaijun Zhou

**Affiliations:** ^1^ Department of Animal Science, University of California Davis, Davis, CA, United States; ^2^ USDA, ARS, USNPRC, Avian Disease and Oncology Laboratory, East Lansing, MI, United States

**Keywords:** transcriptome, annotation, transcript isoform, nanopore, long-read sequencing, chicken

## Abstract

To identify and annotate transcript isoforms in the chicken genome, we generated Nanopore long-read sequencing data from 68 samples that encompassed 19 diverse tissues collected from experimental adult male and female White Leghorn chickens. More than 23.8 million reads with mean read length of 790 bases and average quality of 18.2 were generated. The annotation and subsequent filtering resulted in the identification of 55,382 transcripts at 40,547 loci with mean length of 1,700 bases. We predicted 30,967 coding transcripts at 19,461 loci, and 16,495 lncRNA transcripts at 15,512 loci. Compared to existing reference annotations, we found ∼52% of annotated transcripts could be partially or fully matched while ∼47% were novel. Seventy percent of novel transcripts were potentially transcribed from lncRNA loci. Based on our annotation, we quantified transcript expression across tissues and found two brain tissues (i.e., cerebellum and cortex) expressed the highest number of transcripts and loci. Furthermore, ∼22% of the transcripts displayed tissue specificity with the reproductive tissues (i.e., testis and ovary) exhibiting the most tissue-specific transcripts. Despite our wide sampling, ∼20% of Ensembl reference loci were not detected. This suggests that deeper sequencing and additional samples that include different breeds, cell types, developmental stages, and physiological conditions, are needed to fully annotate the chicken genome. The application of Nanopore sequencing in this study demonstrates the usefulness of long-read data in discovering additional novel loci (e.g., lncRNA loci) and resolving complex transcripts (e.g., the longest transcript for the *TTN* locus).

## Introduction

Chicken (*Gallus gallus domesticus*) is the most widespread domesticated farm animal for egg and meat production, with a total population of 37.2 billion for the year 2020 (http://www.fao.org/). Besides its agronomic importance, chicken has contributed greatly to biological studies on evolution, development, and immunology. In 2004, the first draft whole chicken genome was assembled with an estimated set of 20-23,000 protein-coding genes (PCGs) (Hillier et al., 2004). This effort offered a genome-wide view for understanding the configuration of the chicken genome (∼1.2 × 10^9^ bp), and the evolution of coding and noncoding genes in vertebrate genomes. Additional efforts including high-resolution genetic linkage maps, radiation hybrid maps, targeted genome sequences, allowed us to know the chicken karyotype, which consists of 38 pairs of autosomes and a pair of sex chromosomes (chromosomes W and Z) (Schmid et al., 2015). Since then, continuous efforts have been made to improve the completeness of chicken genome. For instance, Warren et al. (2017) added an additional 183 Mb sequences and assembled chromosomes 30-33 for the chicken reference genome. To fill the gaps of the chicken reference genome, recently two pangenomes were built that reported additional sequences absent from the GRCg6a reference genome (Wang K. et al., 2021; Li et al., 2022).

The functional annotation of the chicken genome is also being produced in parallel. The two most commonly used databases, i.e., Ensembl (https://uswest.ensembl.org) and National Center for Biotechnology Information (NCBI, https://www.ncbi.nlm.nih.gov/) regularly update the chicken genome annotation. For instance, Ensembl release (V102) includes 16,779 PCGs and 39,288 transcripts, representing 2.34 transcripts per gene, which is quite low compared to human with ∼10 transcripts per gene. The high estimate in human can be attributed to several large global efforts, such as GENCODE, which is part of the ENCODE (ENCyclopedia Of DNA Elements) consortium which aims to identify and classify all gene features in human and mouse genomes. In farm animals, likewise, the Functional Annotation of ANimal Genome (FAANG) consortium was formed in order to improve the annotation of livestock genomes (Giuffra et al., 2019; Clark et al., 2020). In prior work, Kern et al. (2021) annotated noncoding genomes of three important livestock species including chicken, and predicted 29,526 regulatory element-gene interactions in chickens. In addition, Kern et al. (2018) identified a total of 9,393 long non-coding RNAs (lncRNAs) (including 5,288 novel lncRNAs) by utilizing short-read transcriptomes from eight chicken tissues.

Transcribed regions, though accounting for only ∼3% of the chicken genome, like in other higher organisms, are very complex due to alternative usage of transcription start sites, splice junctions, and polyadenylation sites. Alternative splicing has been shown to play important roles in evolution, phenotypic diversity, and organ development (Keren et al., 2010; Baralle and Giudice, 2017; Wright et al., 2022). For example, Yu et al. (2019) identified five alternative splice variants of the *TYR* gene associated with skin melanogenesis in chickens. To annotate these features, transcriptome profiling provides important and useful resources (Yandell and Ence, 2012). Jehl et al. (2020) annotated 1,199 and 13,009 additional PCGs and lncRNA genes, respectively, (compared to Ensembl V94) using 364 short-read transcriptomes derived from 25 chicken tissues. In human, a comprehensive annotation using transcriptomes of 41 tissues generated by the Genotype-Tissue Expression (GTEx) Consortium improved transcript prediction for 13,429 genes, including 1,831 (63%) Online Mendelian Inheritance in Man (OMIM) genes and 317 neurodegeneration-associated genes (Zhang et al., 2020). This analysis demonstrates that a detailed annotation enhances our understanding of genome-to-phenome connections. Although short-read sequencing is widely used for annotating human and animal genomes, it cannot accurately assemble and, thus, resolve the complex structure of transcript isoforms.

The contiguity of the long-read sequencing technology can sequence full-length transcripts, therefore, is better suited for dissecting the complexity of transcript structure compared to short-read sequencing (Amarasinghe et al., 2020). Iso-Seq by Pacific Biosciences is one long-read sequencing technology that is widely used in profiling full-length transcriptomes in several species including human (Kuo et al., 2020), pig (Beiki et al., 2019), and rabbit (Chen et al., 2017). In chickens, Thomas et al. (2014) used Iso-Seq and identified 9,221 new transcript isoforms in embryonic chicken heart tissue. Later on, Kuo et al. (2017) annotated 64,277 additional distinct transcripts (55,315 in brain and 9,206 in embryo) using Iso-Seq plus 5′ cap selection in chicken brain and embryo tissues. Despite these pioneering efforts, only a few tissues were studied making it unlikely that the majority of chicken transcript isoforms have been identified.

Oxford Nanopore Technologies has provided an alternative long-read sequencing approach (Amarasinghe et al., 2020), which has been applied in cattle (Halstead et al., 2021), duck (Lin et al., 2021) and many other species, but not yet in chickens. Nanopore long-read sequencing allows for accurate identification and quantification of transcript isoforms and for resolving complex isoforms (Byrne et al., 2017; Soneson et al., 2019; Chen et al., 2021). In this study, we aimed to identify and characterize transcripts in a diverse set of 19 chicken tissues (cerebellum, hypothalamus, cortex, duodenum, jejunum, ileum, cecum, colon, testis, ovary, adipose, gizzard, heart, kidney, liver, lung, muscle, spleen, and thymus) from adult birds using Oxford Nanopore long-read sequencing. The data generated from this study will be a valuable source to improve our understanding of the complexity of the chicken transcriptome, and also aid in efforts to associate gene expression with phenotypic traits.

## Methods and materials

### Sample collection

All animals and samples used in this study were obtained in concordance with Protocol for Animal Care and Use no. 18464 (approved by Institutional Animal Care and Use Committee at the University of California at Davis). All tissues were from one of two FAANG pilot projects (FarmENCODE) (Tixier-Boichard et al., 2021). In brief, highly inbred ADOL experimental White Leghorn lines 6_3_ and 7_2_ were intermated to produce F_1_ progeny, and 4 male and 2 female individuals were euthanized with CO_2_ at 20 weeks of age. After euthanizing, all tissues were immediately collected within 1–2 h and stored at −80°C until further use.

### RNA extraction and library preparation

RNA extraction and library preparation were performed as described in Halstead et al. (2021). In brief, frozen tissues were mashed using a pestle in a mortar filled with liquid nitrogen. Next Trizol reagent (Invitrogen, Carlsbad, CA, United States) was added to extract total RNA using the Direct-zol RNA Mini Prep Plus kit (Zymo Research, Irvine, CA, United States). The integrity and quality of the extracted RNA was checked using an Experion electrophoresis system (Bio-Rad, Hercules, CA, United States) and samples passing quality control were used for library preparation. First, 50 ng of total RNA in a volume of 9 μl was mixed with 1 μl 10 μM VNP primer and 1 μl 10 mM dNTPs, then incubated 5 min at 65°C. The resulting products were used for strand-switching and reverse transcription reactions (Halstead et al., 2021). Then barcodes were ligated to the cDNA products generated from the last step using the Oxford Nanopore PCR barcoding expansion 1-96 kit (cat. no. EXP-PBC096), which were further ligated with adapters from the SQK-DCS109 kit following the manufacturer’s guidelines. Products were loaded onto a PromethION flow cell (vR9.4.1) for sequencing.

### Base calling, quality control, and preprocessing

After base calling and de-multiplexing with the ont-guppy-for-minknow (v3.0.5) tool (https://nanoporetech.com/), NanoPlot (v1.0.0) software was used to summarize read length and average quality, among others. Then, the Pychopper v2 software (https://github.com/nanoporetech/pychopper) was employed to identify and orient full-length reads, which were mapped to reference genomes (GRCg6a, Ensembl V102) with options of “-ax splice -uf -k14 -G 1000000” using the minimap2 software (Li, 2018). We discarded chimeric and multi-mapped reads, as well as reads with a minimum quality score of 10 using SAMtools (v1.9) (Li et al., 2009). The HTSeq 0.13.5 software (Anders et al., 2015) was used for summarizing read counts of genes, which were further normalized using the “variance stabilizing transformation (VST)” function with the DEseq2 software tool (Love et al., 2014). Principal component analysis (PCA) based on normalized read counts was carried out using the “plotPCA” function of the DEseq2 (Love et al., 2014).

### Reference-guided prediction of transcript isoforms

To predict transcripts, we used a computational pipeline supported by the Oxford Nanopore Technology community (https://github.com/nanoporetech/pipeline-nanopore-ref-isoforms). Briefly, the oriented full-length reads with fastq format were pooled together and then mapped to the Ensembl annotation (GRCg6a, V102) using minimap2 (Li, 2018) in order to carry out a reference-guided transcriptome assembly. Before performing transcript assembly, we predicted the length of the poly A tail using the PolyAtailor tool (Liu et al., 2022). Then, mapped reads were used to annotate transcripts using the StringTie2 software (Kovaka et al., 2019) in the long-read mode (with the option of “-L”). Transcripts on unplaced scaffolds, as well as those with exon coverage <100% and read depth <2 were excluded. Only single-exon transcripts with expression TPM >1 in >2 samples of a tissue, and multi-exon transcripts with expression TPM >0.1 in >2 samples of a tissue were retained. Finally, we excluded transcripts categorized as potential artifacts (see the *Comparing predicted transcripts to previous annotations* section).

### Prediction of coding and non-coding transcripts and loci

To predict the coding potential of predicted transcripts, we employed TransDecoder (https://github.com/TransDecoder/TransDecoder) and CPP2 (Kang et al., 2017). After prediction of coding potential, the list of non-coding transcripts was obtained, which was used to predict whether they are lncRNA loci using FEElnc (Wucher et al., 2017).

### Comparing predicted transcripts to previous annotations

The predicted transcripts were compared to the Ensembl (V102) and NCBI reference (V105) annotations using GffCompare (version 0.11) (Pertea and Pertea, 2020) and classified into 14 classes. According to Halstead et al. (2021), the predicted transcripts could be grouped into four categories: exact match (class code “=”), which means the intron chains of our annotated transcripts exactly matched reference annotations; novel isoform (class codes ‘c,’ ‘k,’ ‘j,’ ‘m,’ ‘n,’ or ‘o’), which means predicted transcript did not match a reference transcript but could match a reference gene; novel loci (class codes ‘i,’ ‘u,’ ‘y,’ or ‘x’), which means the predicted transcript did not match either a reference transcript or a reference locus; and potential artifacts (class codes ‘e,’ ‘s,’ or ‘p’), which are possibly due to mapping error, e.g., pre-mRNA fragments, polymerase run-on, etc. To compare our prediction with novel transcripts reported by Thomas et al. (2014), we first converted positions of their transcripts from galGal4 to GRCg6a using the liftover software (Kuhn et al., 2013). Then the GffCompare tool was used for comparing our annotation to their transcripts (Pertea and Pertea, 2020).

### Quantification of predicted transcripts

We extracted sequences of predicted transcripts using GffRead v0.12 (Pertea and Pertea, 2020), which constituted a reference transcriptome in the FASTA format. Then, we mapped full-length reads generated by Pychopper (https://github.com/nanoporetech/pychopper) to the predicted transcriptome using minimap2 (v2.1) (Li, 2018). The transcript expression was quantified using Nanocount (v0.2.4) (Leger, 2020). Based on the metric of the transcripts per million (TPM), we categorized transcripts as highly (average TPM >10), moderately (1 < average TPM ≤10), or lowly expressed (average TPM ≤1) (Halstead et al., 2021).

### Tissue-specificity analysis

The tissue specificity of transcripts expression across tissues were evaluated by using a tissue specificity index (*TSI*) (Julien et al., 2012; Halstead et al., 2021):

$$TSI=\frac{\max_{1\leq i\leq n}\left(x_{i}\right)}{\overset{n}{\underset{i=1}{\sum }}x_{i}}$$

where *x*_*i*_ is an average of transcript expression (TPM) in a given tissue, n is the number of tissues. Transcripts were then categorized as tissue-specific (TSI ≥0.8), broadly expressed (TSI <0.5), or biased towards a group of tissues (0.5 ≤ TSI <0.8). To reveal functional biology of tissue-specific transcripts, we extracted tissue-specific transcript sequences and aligned them to the SwissProt (protein sequence database, V5) using the Diamond blastx tool (v2.0.11.149) (Buchfink et al., 2015). Next, we then carried out functional enrichment (only considering Gene Ontology Biological Process terms) using the matched UniProt identifiers via the PANTHER tool (Mi et al., 2013). The false discovery rate (FDR) approach (Benjamini and Hochberg, 1995) was used for multiple testing corrections and FDR value less than 0.05 was set as the significance threshold.

### Differential alternative splicing analysis

To detect differential alternative splicing (DAS) events, we employed the LIQA software (Hu et al., 2021). Based on our annotation, we quantified isoform expression using the “quantify” function. Then DAS events between tissues were detected using “diff” within the LIQA tool (Hu et al., 2021). After multiple testing correction (Benjamini and Hochberg, 1995), the threshold of significance was set as FDR <0.05.

## Results

**FIGURE 1:**
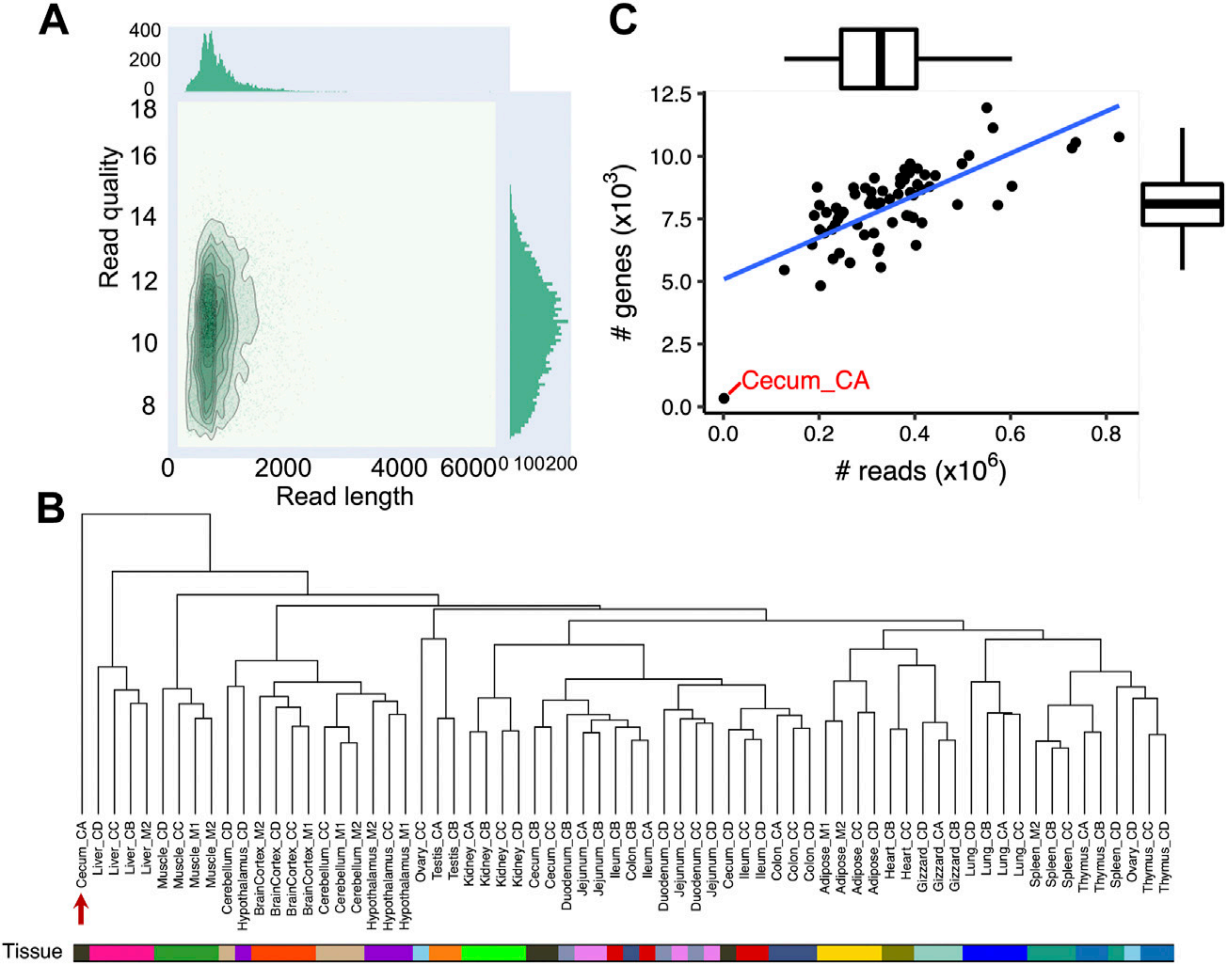
Data summary of 68 chicken Nanopore long-read transcriptome datasets. **(A)** Bivariate plot (De Coster et al., 2018) depicting read length (x-axis) and quality (y-axis) of Nanopore long-read transcriptome reads **(B)** Hierarchical clustering of 68 chicken Nanopore long-read transcriptome samples used in this study. The dendrogram is built based on gene expressions quantified with transcripts per million (TPM ≥0.1). The distance between individuals is indicated by 1-r, where r is the Pearson correlation coefficient. The red arrow indicates sample Cecum_CA, which did not cluster with other cecal samples. **(C)** Correlation between the number of sequencing reads (x-axis) and the number of expressed genes (y-axis, TPM >0.1). The Pearson’s correlation is 0.71 (*p* = 1.30 × 10^−11^).

To annotate transcripts of the chicken genome, we sequenced 68 samples that covered 19 different and diverse tissues collected from six individual adult White Leghorn birds (two females: CC and CD; and four males: CA, CB, M1, M2) (Supplementary Table S1). The 19 tissues collected were cerebellum, hypothalamus, cortex, duodenum, jejunum, ileum, cecum, colon, testis, ovary, adipose, gizzard, heart, kidney, liver, lung, muscle, spleen and thymus. Long-read sequencing generated a total of 23.8 million reads, with an average of 344,650 reads per tissue and average length of 790 bases (Figure 1A; Supplementary Table S2).

PCA and hierarchical clustering of mapped sequencing reads to the Ensembl annotation (GRCg6a, version 102) revealed that samples generally clustered according to the origin of tissue as expected (Figure 1B; Supplementary Figure S1; Supplementary Table S3). Moreover, we found samples from the same biological system tended to cluster together, such as brain cortex, cerebellum and hypothalamus from the central neural system; and cecum, colon, duodenum, ileum, and jejunum from the intestinal system (Figure 1B). The one exception was Cecum_CA as seen in both the PCA plot and hierarchical clustering (indicated by the red arrow in Figure 1B; Supplementary Figure S1). Overall, there was a strong correlation between the number of sequenced reads and the number of expressed genes (Pearson’s correlation = 0.71, *p* = 1.30 × 10^−11^) with the exception of Cecum_CA, which had extremely low number of sequencing reads (1,279), suggesting the unexpected clustering is possibly due to insufficient sequencing depth (Figure 1C). Further analysis indicated that 672 out of 1,003 full-length reads from Cecum_CA could align to the GRCg6a genome, corresponding to a mapping rate of 67%, while the average mapping rate of the remaining samples is 94.3%. In the light of these findings, we excluded Cecum_CA in all further downstream analyses.

**FIGURE 2:**
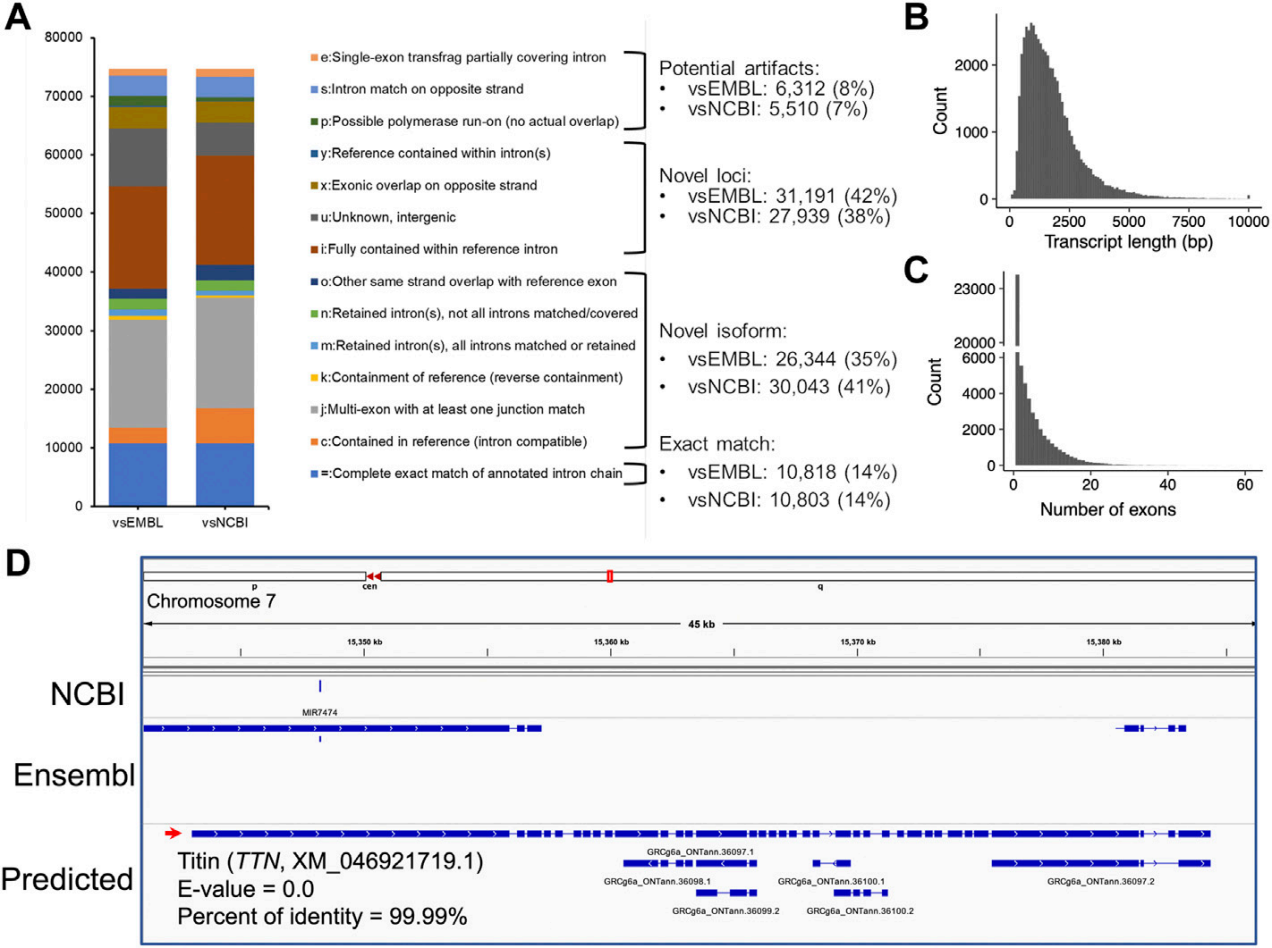
Transcript assembly using Nanopore long-read transcriptome data. **(A)** Comparisons of predicted transcripts against Ensembl (V102, vsEMBL) and NCBI annotation (V105, vsNCBI). The transcripts were classified according to the GffCompare software (Pertea and Pertea, 2020). The panels **(B,C)** depict the distributions of predicted transcript length and exon numbers, respectively. **(D)** A screenshot showing the predicted longest transcript, which is located on chromosome 7 (15,343,033-15,384,347). Blast analysis indicated the transcript matched to the *TTN* gene locus encoding the titin protein.

To assemble potential transcripts, we identified, oriented, and trimmed full-length reads using Pychopper v2. Further analysis indicated that all full-length reads had poly(A) tails with average length of 19 bases (range was 8-637 bases) (Supplementary Figure S2). Then, StringTie in the long read mode was used to predict transcripts (https://github.com/nanoporetech/pipeline-nanopore-ref-isoforms). As a result, 79,757 transcripts in 54,551 loci were identified. After filtering out transcripts on unplaced scaffolds, as well as those with exon coverage <100% and read depth <2, we obtained 74,665 transcripts in 50,569 loci, of which there were 45,132 multi-exon and 29,533 single-exon transcripts. Moreover, we required multi-exon transcripts with expression TPM >0.1 and single-exon transcripts with expression TPM >1 in at least 2 samples of a tissue. By doing so, 61,556 transcripts in 45,284 loci were remained. To further exclude potential artifacts, we compared assembled transcripts with NCBI (V105) and Ensembl (V102) reference annotations. The results are shown in Figure 2A and Table 1. Overall, we found ∼14% of predicted transcripts exactly matched the reference annotations (Figure 2A). With the Ensembl annotation, 77% of them were considered as novel transcripts, either novel isoforms (35%) or novel loci (42%). In addition, ∼8% were potential artifacts, possibly caused by pre-mRNA fragments, polymerase run-on, or mapping errors (Figure 2A; Table 2). After excluding these potential artifacts, we kept 55,382 transcripts in 40,547 loci, representing ∼1.4 transcripts per locus (Supplementary Data S1).

**TABLE 1:** Comparison of reference and predicted transcripts using GffCompare tool.

	Predicted vs. Ensembl		Predicted vs. NCBI		NCBI vs. Ensembl	
Level	Sensitivity	Precision	Sensitivity	Precision	Sensitivity	Precision
Base	70.9	30.6	54.1	41.3	86.6	43.6
Exon	62.6	55.3	55.1	55.6	78.5	64.5
Intron	66.3	74.2	58.8	77.5	88.6	72.2
Transcript	38.7	14.5	21.1	14.5	41.6	21.1
Locus	57.8	17.5	54.3	17.0	59.7	47.3
Missed exons	44,538/179919 (24.8%)		60,304/211468 (28.5%)		10,378/202,369 (5.1%)	
Novel exons	63,322/201,393 (31.4%)		54,465/201,393 (27.0%)		50,528/252,210 (20.0%)	
Missed introns	41,164/157,463 (26.1%)		53,133/185,508 (28.6%)		6,790/175,889 (3.9%)	
Novel introns	22,985/140,865 (16.3%)		19,416/140,865 (13.8%)		30,813/215,950 (14.3%)	
Novel loci	32,725/50,569 (64.7%)		29,332/50,569 (58.0%)		5,656/23,336 (24.2%)	

The annotation versions of NCBI and Ensembl are V105 and V102, respectively.

**TABLE 2:** Number of transcripts as a function of Gffcompare codes.

Code^a^	Description	Category	Number of predicted transcripts^b^	Number of filtered transcripts^b^
=	Complete, exact match of intron chain	Exact match	10,818	9,207
c	Contained in reference (intron compatible)	Novel isoforms	2,596	2,150
k	Containment of reference (reverse containment)	Novel isoforms	627	551
m	Retained intron(s), all introns matched or retained	Novel isoforms	1,119	980
n	Retained intron(s), all introns matched/covered	Novel isoforms	1,832	1,520
j	Multi-exon with at least one junction match	Novel isoforms	18,460	13,666
e	single exon transfrag partilly covering an intron, possible pre-mRNA fragment	Potential artifacts	1,138	—
o	Other same strand overlap with reference exons	Novel isoforms	1,710	1,362
s	Intron match on the opposite strand (likely a mapping error)	Potential artifacts	3,479	—
x	Exonic overlap on the opposite strand (like o or e but on the opposite strand)	Novel loci	3,683	2,807
i	Fully contained within a reference intron	Novel loci	17,495	15,782
y	Contains a reference within its intron(s)	Novel loci	193	134
p	Possible polymerase run-on (no actual overlap)	Potential artifacts	1,695	—
u	None of the above (unknown, intergenic)	Novel loci	9,820	7,223
		Total transcripts	74,665	55,382

a The explanation of Gffcompare codes is retrieved from https://ccb.jhu.edu/software/stringtie/gffcompare.shtml.

b The number of transcripts were summarized according to the comparing results of the Gffcompare with Ensembl (V102) annotation.

The length of predicted transcripts ranged from 49 to 34,500 bases, with a mean length of 1,767 bases (Figure 2B). The longest transcript is located on chromosome 7 (15,343,033-15,384,347), and matched the *TTN* gene encoding the giant protein titin (NCBI reference sequence XM_046921719.1, E-value = 0.0, percent of identity = 99.99%) (Figure 2D). This protein plays an important role in skeletal muscle movement, but its gene locus has not been annotated in both NCBI (V105) and Ensembl (V102) GRCg6a references (Figure 2D). Moreover, we found the annotated 55,382 transcripts are supported by 171,651 unique exons, with an average estimate of 4.34 exons per transcript (Figure 2C).

To determine the coding potential of the predicted transcripts, we employed CPC2 and TransDecoder. The former predicted 21,984 transcripts at 12,999 loci with coding potential, and the latter one predicted open reading frames for 30,727 transcripts corresponding to 19,306 loci. In total, we predicted 30,967 uniquely potential coding transcripts at 19,461 loci, representing 1.6 transcripts per locus (Supplementary Table S4). Furthermore, we surveyed whether the remaining 24,415 transcripts were lncRNAs. To do so, we employed the FEELnc and found 16,495 potential lncRNA transcripts at 15,512 loci (Supplementary Table S4).

**FIGURE 3:**
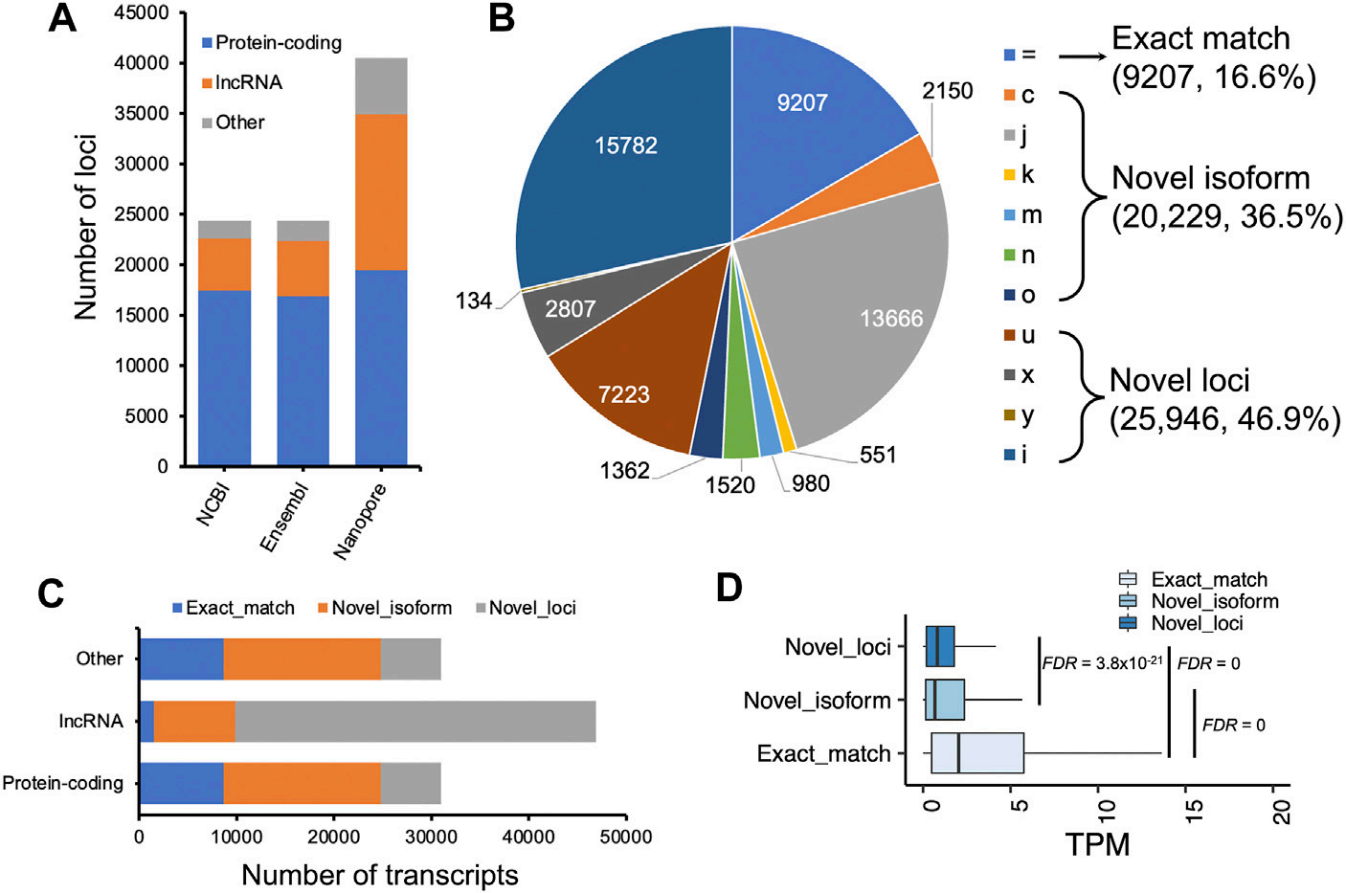
Characterization of assembled transcripts. **(A)** Number of loci in NCBI (V105), Ensembl (V102) and our annotations. **(B)** Pie chart depicting GffCompare types to Ensembl annotation (V102). **(C)** Number of transcripts as a function of protein-coding, lncRNA, and other non-coding loci. **(D)** Transcript expression measured as transcript per million (TPM) as a function of different types of transcripts classified by GffCompare tool. Exact match: GffCompare code “=”, which means the intron chains of our annotated transcripts can exactly match to reference annotations; Novel isoform: GffCompare codes ‘c,’ ‘k,’ ‘j,’ ‘m,’ ‘n,’ or ‘o’, which means predicted transcript cannot match a reference transcript but can match a reference gene; novel loci: GffCompare codes ‘i,’ ‘u,’ ‘y,’ or ‘x’, which means predicted transcript cannot match either a reference transcript or a reference locus. The type ‘y’ only has 134 transcripts, a small proportion that is not visible in the pie chart. Student’ t tests were carried out between two groups of transcripts, and *p* values were adjusted by using false discovery rate (FDR) method (Benjamini and Hochberg, 1995).

We compared our predictions to two reference annotations and found the number of transcripts per locus of our annotation (∼1.4) was lower compared to both reference annotations (Ensembl v102: ∼1.8 transcripts per locus; NCBI v105: ∼3.3 transcripts per locus), but we predicted ∼20,000 more loci, of which a substantial proportion are lncRNA loci (Figures 3A,C). At the transcript level, we classified transcripts into three categories: 1) exact match: predicted transcripts completely matched to reference annotations; 2) novel isoform: predicted transcripts did not match reference transcripts but matched reference loci; 3) novel loci: predicted transcripts did not match any reference loci or transcripts (Figure 3B). Concordantly, we found our prediction identified a high proportion of “novel loci” transcripts (47%), followed by “novel isoforms” (37%) when compared to the Ensembl annotation (V102) (Figure 3B). A similar pattern was observed when compared to the NCBI annotation (Supplementary Figure S3). By further comparing lncRNA loci predicted in this study with those predicted by Jehl et al. (2020), we found ∼83% of our predicted lncRNA transcripts matched their annotations (Supplementary Figure S4). Thomas et al. (2014) also generated 1,849,786 cDNA sequencing reads that identified 9,221 novel transcripts in embryonic chicken heart using Pacific Biosciences long-read technology. However, the unavailability of their full annotation prevented us to make a complete assessment, but when comparing their available novel transcripts with our annotation, we found 89% of them completely or partially matched. There are still 1,000 transcripts categorized as “novel loci” (Supplementary Figure S5). Moreover, we found the transcripts grouped into the “novel isoform” and “novel loci” categories tended to be lowly expressed, while the expressions of transcripts in “exact match” group are significantly higher (Student’s t-test, FDR <0.01, Figure 3D).

**FIGURE 4:**
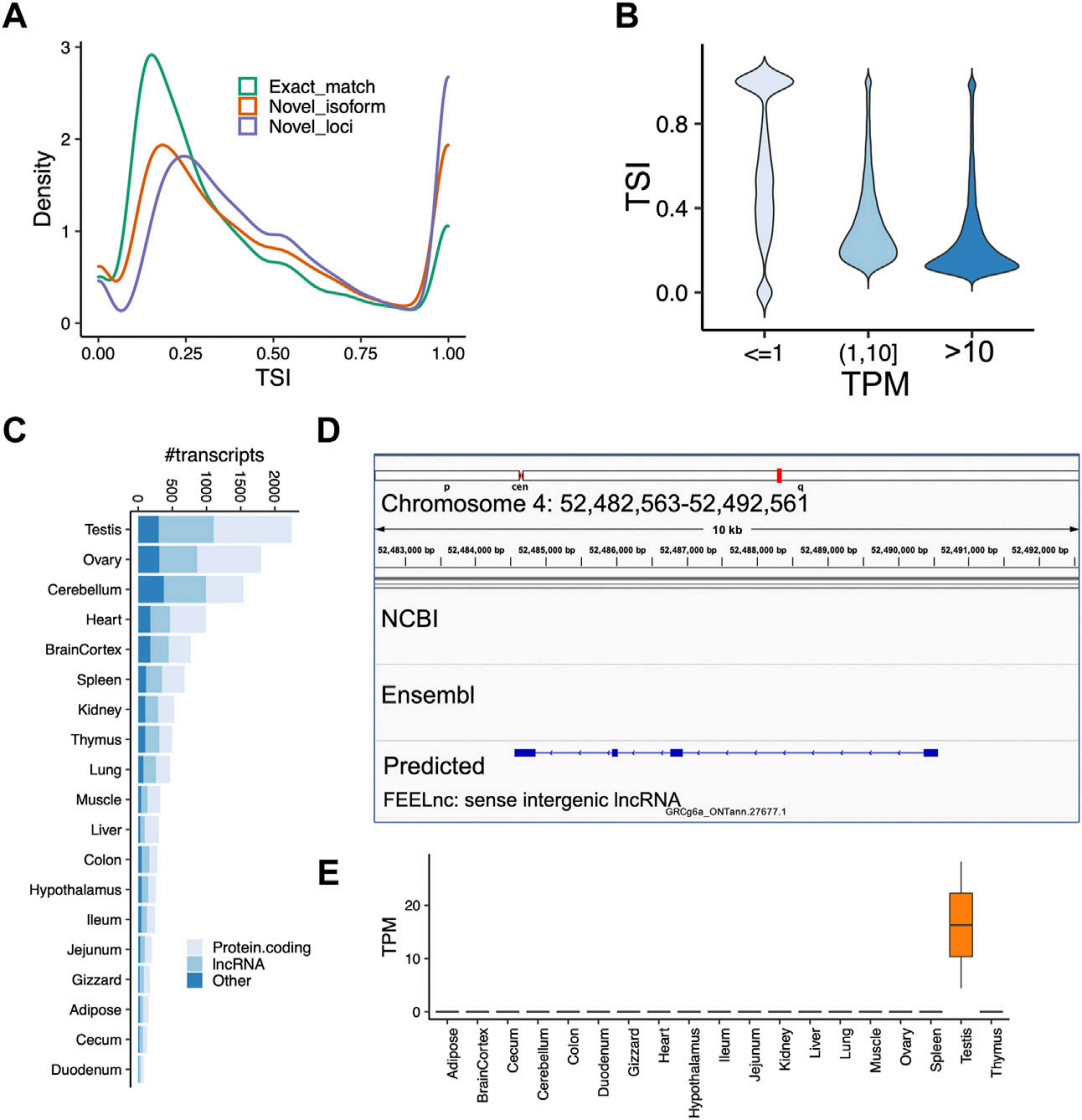
Analysis of tissue-specificity across tissues. **(A)** Tissue specificity index (TSI) as a function of different types of transcripts classified by GffCompare. Code “ = ” means the intron chains of our annotated transcripts can exactly match to reference annotations (Exact match); Codes ‘c,’ ‘k,’ ‘j,’ ‘m,’ ‘n,’ or ‘o’ mean predicted transcript cannot match a reference transcript but can match a reference gene (Novel isoform); Codes ‘i,’ ‘u,’ ‘y,’ or ‘x’ means predicted transcript cannot match either a reference transcript or a reference locus (novel loci). **(B)** Transcript expression measured as transcript per million (TPM) as a function of TSI. We grouped transcripts according to their expressions. **(C)** Number of tissue-specific transcripts in each tissue. **(D)** A screenshot showing a novel transcript only predicted by our data, which is located on chromosome 4 (52,482,563–52,492,561). **(E)** TPM expressions of the predicted lncRNA transcript shown in the panel **(D)**. The transcript is highly expressed in testes samples, but not any other tissue. The FEELnc predicted it as a sense intergenic lncRNA.

**FIGURE 5:**
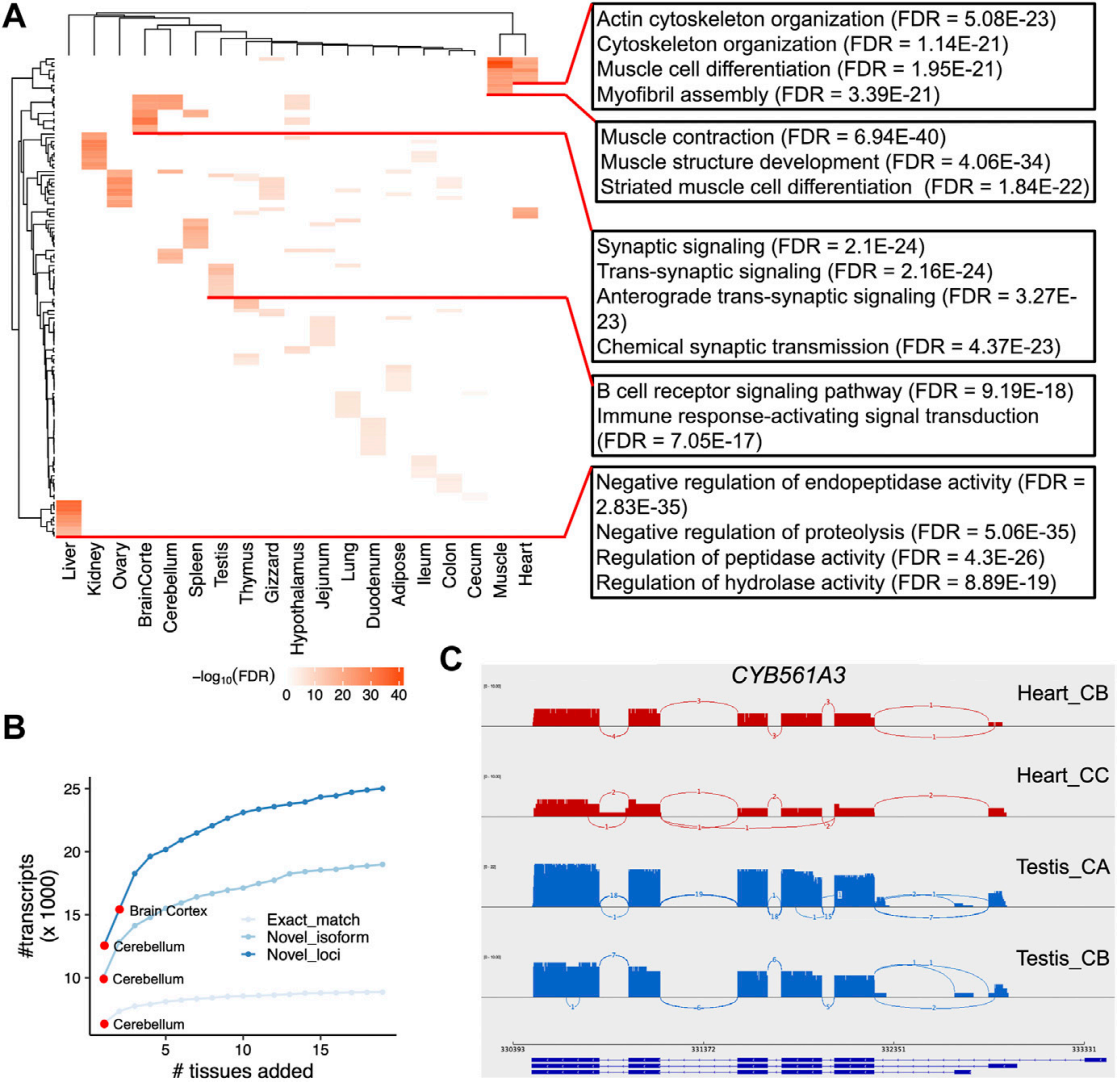
Functional enrichment of tissue-specific transcripts and differential alternative splicing analysis. **(A)** Heatmap depicting the negative log_10_FDR (false discovery rate) values for the top 10 Gene Ontology (GO) Biological Process terms. At the right side, we show several examples of GO terms, as well as their FDR values. **(B)** Number of unique transcripts detected as a function of tissues added. Transcripts are categories into three types (see Methods). **(C)**. Sashimi plots of *CYB561A3* gene that showed DAS between heart (red) and testis (blue).

Next, we sought to identify tissue-specific transcripts. By quantifying transcript expressions, we found the number of expressed transcripts and loci ranged from 14,841 (liver) to 28,648 (cerebellum), and from 10,285 (liver) to 21,662 (cerebellum), respectively (Supplementary Figure S6). The tissue specificity index (TSI) indicated that the set of “exact match” transcripts tended to be lowly tissue-specific, while “novel isoform” and “novel loci” transcripts are highly tissue-specific (Figure 4A). We observed that the set of transcripts with low expression tended to have high tissue-specificity, while in contrast, highly expressed transcripts are commonly found across many tissues (Figure 4B). Moreover, we identified tissue-specific transcripts and found the reproductive tissues (i.e., testis and ovary) have a high proportion of tissue-specific transcripts, followed by two brain-related tissues (i.e., cerebellum and cortex) (Figure 4C). For instance, we identified a novel transcript located on chromosome 4 (52,482,563-52,492,561), which is specifically expressed in the testes samples (Figures 4D,E). This transcript was predicted as a sense intergenic lncRNA by the FEELnc software (Wucher et al., 2017) (Supplementary Tables S4, S5). By aligning sequences of tissue-specific transcripts to SwissProt (V5) (Buchfink et al., 2015) and carrying out functional enrichment analysis with PANTHER (Mi et al., 2013), we found that tissue-specific transcripts recapitulated tissue biology (Figure 5A; Supplementary Table S6), e.g., muscle contraction, muscle cell differentiation enriched in muscle and heart tissues, trans-synaptic signaling and nervous system development in cerebellum and brain cortex, and B cell receptor signaling pathway in spleen (Figure 5A; Supplementary Table S6), a finding concordant with previous results (Yang et al., 2018; Fang et al., 2020).

The utilization of large scale of tissues allowed us to investigate which tissue is best able to capture the most transcripts and annotate the chicken genome. Herein we tried to detect the number of unique transcripts expressed as a function of more tissues added. By doing so, we found brain-related tissues (i.e., cerebellum and cortex) could detect higher number of transcripts as expected (Figure 5B; Supplementary Table S7). In addition, our design that includes a diverse set of 19 chicken tissues offers the opportunity to analyze DAS events between chicken tissues. To do so, we quantified isoform expression and identified differential alternative splicing events using LIQA (Hu et al., 2021). As shown in Supplementary Figure S7 and Supplementary Table S8, we found 4,211 loci showing DAS events between tissues (FDR <0.05). For instance, the most significant locus is the *CYB561A3* gene showing DAS between heart and testis (FDR = 9.12 × 10^−16^, Figure 5C). This gene encodes cytochrome B561 family member A3 whose functions are related to cellular iron homeostasis and mitochondrial respiration (Wang et al., 2021).

## Discussion

A well-annotated chicken genome is essential in associating genetic variation to phenotypic variation, and there are a number of ongoing efforts through the Functional Annotation of Animal Genomes (FAANG) consortium (Andersson et al., 2015), which primarily focuses on non-coding functional elements in farm animals including chicken (Kern et al., 2021). In this study, using Oxford Nanopore long-read sequencing on 68 samples from 19 different chicken tissues, we initially annotated 79,757 transcripts in 54,551 loci, while the subsequent filtering resulted in the exclusion of ∼2,000 transcripts. Though all reads used for transcript assembly were predicted to have poly A tails, we also had TPM expression of multi-exon transcripts >0.1, a threshold widely used in the human GTEx project that is robust and sensitive for lowly-expressed genes (Gu et al., 2022; The GTEx Consortium, 2020). Since detection of single-exon transcripts is error-prone, we used a more stringent threshold (TPM >1 in at least two samples of a tissue). Finally, our prediction resulted in the identification of 55,382 filtered transcripts derived from 40,547 loci, representing ∼1.4 transcripts per locus, an estimate lower than the Ensembl (∼1.8 transcripts per locus), and the NCBI annotations (∼3.3 transcripts per locus). The lower estimate in our study might be due in part to the higher number of annotated loci (N = 40,547), i.e., around 2.6-fold higher than both reference annotations.

The number of predicted loci with a transcript in this study was substantially higher than two reference annotations (Esembl V102: 27,955 transcripts in 15,305 loci; NCBI V105: 51,222 in 15,706 loci), while our prediction is lower than Kuo et al. (2017) who annotated 60,000 transcripts and 29,000 genes using the Pacific Biosciences Iso-Seq approach. Unfortunately, the unavailability of their annotation hindered us to make a full comparison. Specifically, we predicted a higher proportion of lncRNA loci, indicating that reference annotations did not annotated lncRNAs well. Indeed, Jehl et al. (2020) annotated an additional 13,009 lncRNA genes (compared to Ensembl V94) using 364 chicken short-read transcriptomes derived from 25 tissues. Furthermore, when we compared our lncRNA transcripts to Jehl et al. (2020), we found over 80% of them completely or partially matched. Still, our annotation contains 4,953 additional novel lncRNA transcripts in spite of using the lncRNA prediction tool FEELnc (Wucher et al., 2017), which was also used by Jehl at al. (2020). This increase might be due to the higher sensitivity of long-read sequencing (Lagarde et al., 2017). Moreover, we found >89% of novel transcripts reported by Thomas et al. (2014) could match our prediction. These results collectively suggest that our annotations are reliable.

Compared to the reference annotations, we observed a higher percentage of novel loci (∼47%) compared to a parallel effort in cattle (Halstead et al., 2021) where 6% of the predicted transcripts did not match to a reference gene). Also, the exact matched transcripts predicted in this study were lower (16% in our study vs. 21% in cattle) though the cattle study did include more tissues (32 in total). Potential reasons for these differences are low number of samples, possible degradation of RNA, or low sequence depth. We also cannot rule out the possibility that the annotation of the bovine reference genome is better compared to the one for chicken. It should be noted that a substantial proportion of novel loci predicted by us are lncRNA with many matching a previous study (Jehl et al., 2020). These results suggest more efforts for annotating the chicken genome are needed in the future. Improved annotation remains even true for the human genome where a recent study found that 36.4% of full-length transcripts were classified as “novel” in the human cortex (Leung et al., 2021). Similarly, another study also reported 17%–55% novel isoforms in human breast cancer samples (Veiga et al., 2022). These studies, together with ours, indicate long-read sequencing is a superior approach for discovering novel isoforms and annotating genomes.

The number of transcripts reported by this study, other studies, and reference genome annotations varies widely, ranging from 27,955 to 74,665. One possible explanation is differences in sequencing depth. Our study generated on average 300,000 reads per sample, ranging from 99,798 (Spleen_CD) to 686,752 (Spleen_CC), while Kuo et al. (2017) generated 805,606 reads in brain and 247,626 reads in embryo. Another possible interpretation is that the number of detectable transcripts is tissue-dependent. Indeed, our study with similar sequencing depth also detected variable number of expressed transcripts across tissues, ranging from 14,841 (liver) to 28,648 (cerebellum). These observations suggest that surveying as many diverse tissues as possible will aid in the detection of tissue-specific transcripts and, thus, better annotate the genome of interest. It is reported that brain tissues have a higher level of alternative splicing, such as skipped exons, alternative 3′ splice site exons, or 5′ splice site exons (Yeo et al., 2004; Melé et al., 2015). Our analysis supports this notion, suggesting brain-related tissues are better for annotating an animal genome. The consistent pattern of the higher number of transcripts observed in brain possibly reflects the complexity of the tissue biology (Naumova et al., 2013; Fang et al., 2020). Moreover, whole embryo is also expected to contain many transcripts since it contains all organs. Unfortunately, our study design did not include the whole embryo, but in the Kuo et al. study (2017), this expectation was not found as 55,932 transcripts were identified in the brain while only 9,368 transcripts were revealed in the embryo.

Previous reports (Sims et al., 2014; Su et al., 2014; Oikonomopoulos et al., 2020) have estimated the power of long and short read RNA sequencing, e.g., Nanopore sequencing needs 40-fold less reads. Based on this estimate, at least 7.5 million long-reads are required per sample, which is becoming more economically feasible given the continued decline in sequencing costs across all platforms. Our study generated ∼300,000 reads per sample, indicating our study likely missed a proportion of lowly expressed transcripts due to the low sequencing depth. This interpretation is also reflected where each gene in our study only produced ∼1.4 transcripts per locus, while each human gene is annotated with ∼10 isoforms (Mathur et al., 2019). In closing, although our study annotated a substantial proportion of novel transcripts, as pointed out earlier, future efforts such as pursuing additional developmental stages and deeper sequencing of transcriptomes are required to fully annotate the chicken genome.

## Data Availability

The datasets presented in this study can be found in online repositories. The names of the repository/repositories and accession number(s) can be found below: https://www.ncbi.nlm.nih.gov/, PRJNA671673. The code used in this study can be found in https://github.com/guandailu/nanopore_annotation.

## Supplementary material

SUPPLEMENTARY DATA S1 Predicted transcripts in the General Feature Format (GTF) format.

SUPPLEMENTARY TABLE S1 Information about tissue sampling used in this study.

SUPPLEMENTARY TABLE S2 Summary statistics of sequencing samples.

SUPPLEMENTARY TABLE S3 Eigenvalues of the top two principal components (PCs) shown in Supplementary Figure 1.

SUPPLEMENTARY TABLE S4 Predicted transcript types (including protein-coding, lncRNA and other non-coding).

SUPPLEMENTARY TABLE S5 A list of tissue-specific transcripts.

SUPPLEMENTARY TABLE S6 Functional enrichment of tissue-specific transcripts (only Biological Process of Gene Ontology terms).

SUPPLEMENTARY TABLE S7 Number of unique transcripts detected when adding more tissues.

SUPPLEMENTARY TABLE S8 A list of loci showing differential alternative splicing (DAS) events between tissues.
