# Supplementary material for: Prediction of transcript isoforms in 19 chicken tissues by Oxford Nanopore long-read sequencing
Source: Front Genet. 2022 Oct 3;13:997460. doi: 10.3389/fgene.2022.997460 (PMC9561881; doi:10.3389/fgene.2022.997460)
Supplement: Supplementary file 1 [file DataSheet2.pdf]

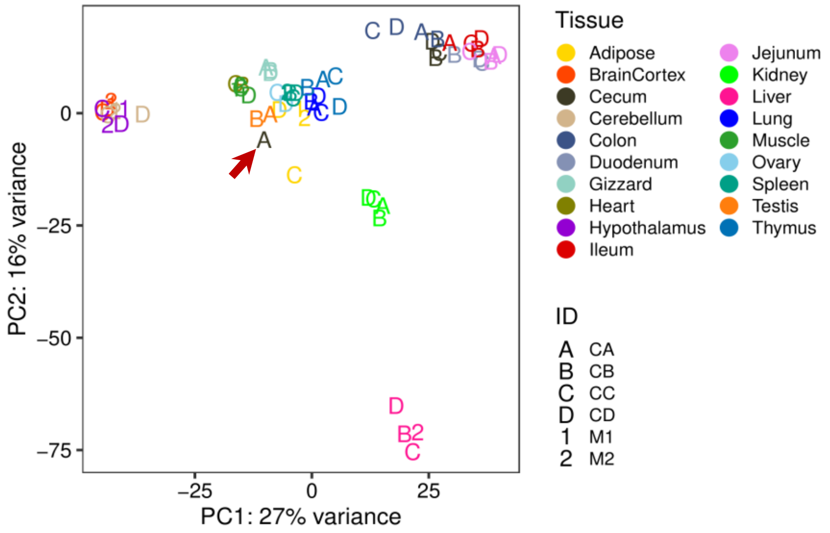

Supplementary Figure 1. Principal component analysis of 68 chicken Nanopore long-read transcriptomes. The red arrow indicated the sample, Cecum_CA, which was not clustered with other samples from the cecum tissue. The corresponding eigenvalues are shown in Supplementary Table 3.

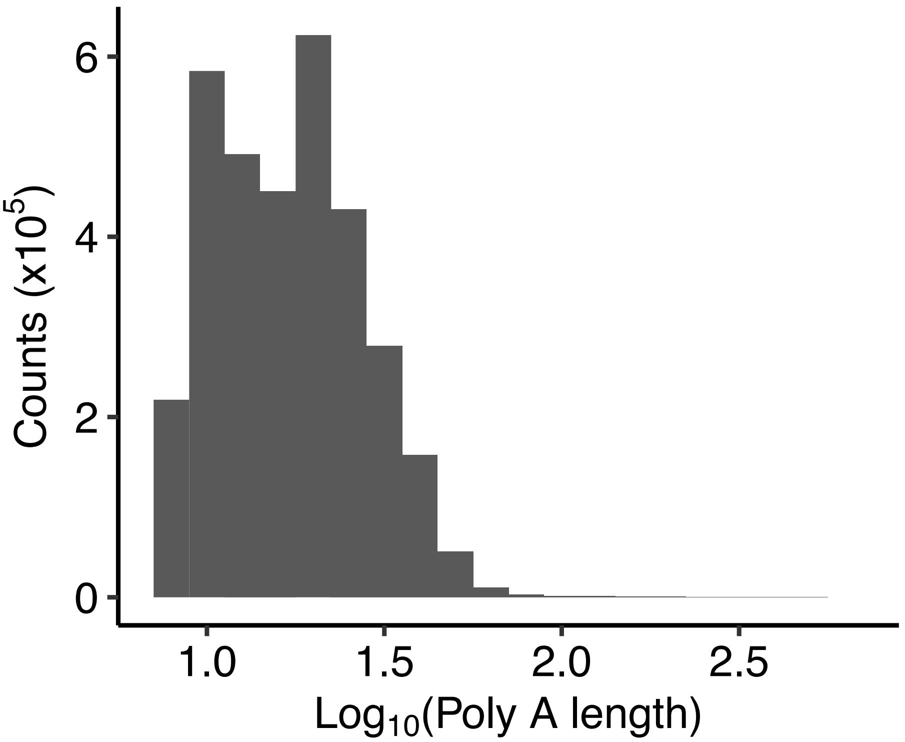

Supplementary Figure 2. Histogram depicting the distribution of poly(A) tail length (bp) predicted by the PolyAtailor software tool (Liu et al., 2022).

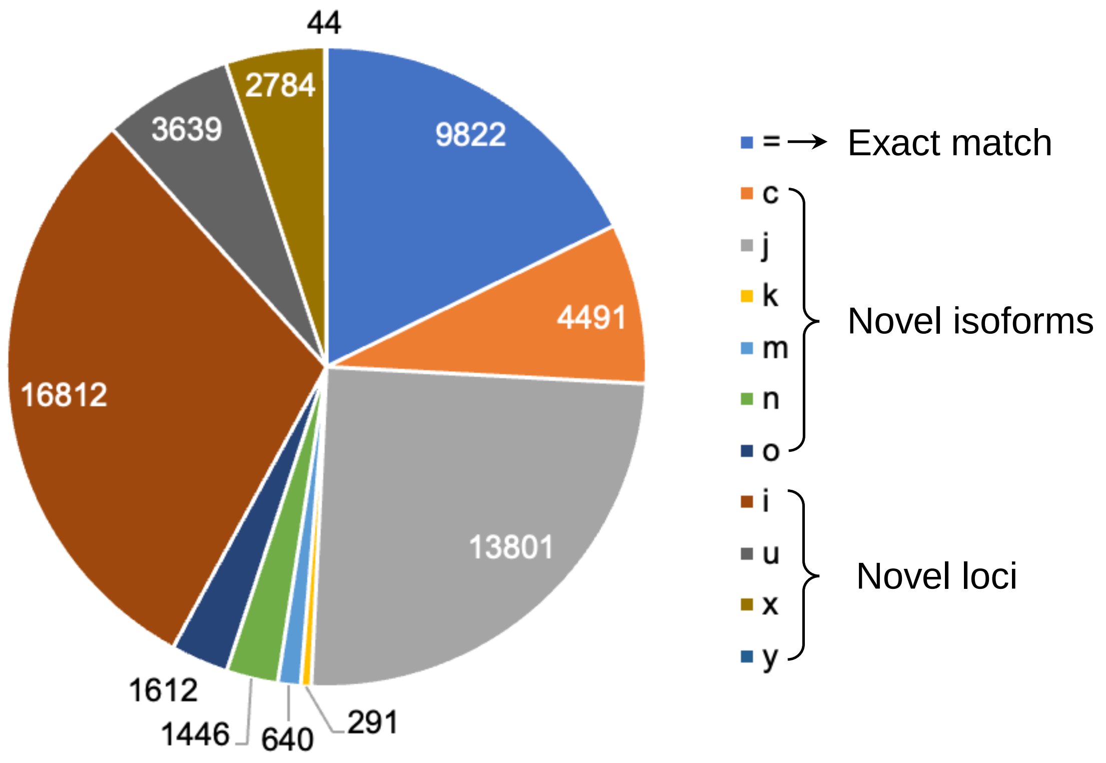

Supplementary Figure 3. GffCompare types when comparing our predicted transcripts to NCBI annotation (V105). Transcripts were classified into: 1) Exact match: GffCompare code "=", which means the intron chains of our annotated transcripts can exactly match to reference annotations; 2) Novel isoform: GffCompare codes 'c,' 'k,' 'j,' 'm,' 'n,' or 'o', which means predicted transcript cannot match a reference transcript but can match a reference gene; 3) Novel loci: GffCompare codes 'i,' 'u,' 'y,' or 'x', which means predicted transcript cannot match either a reference transcript or a reference locus.

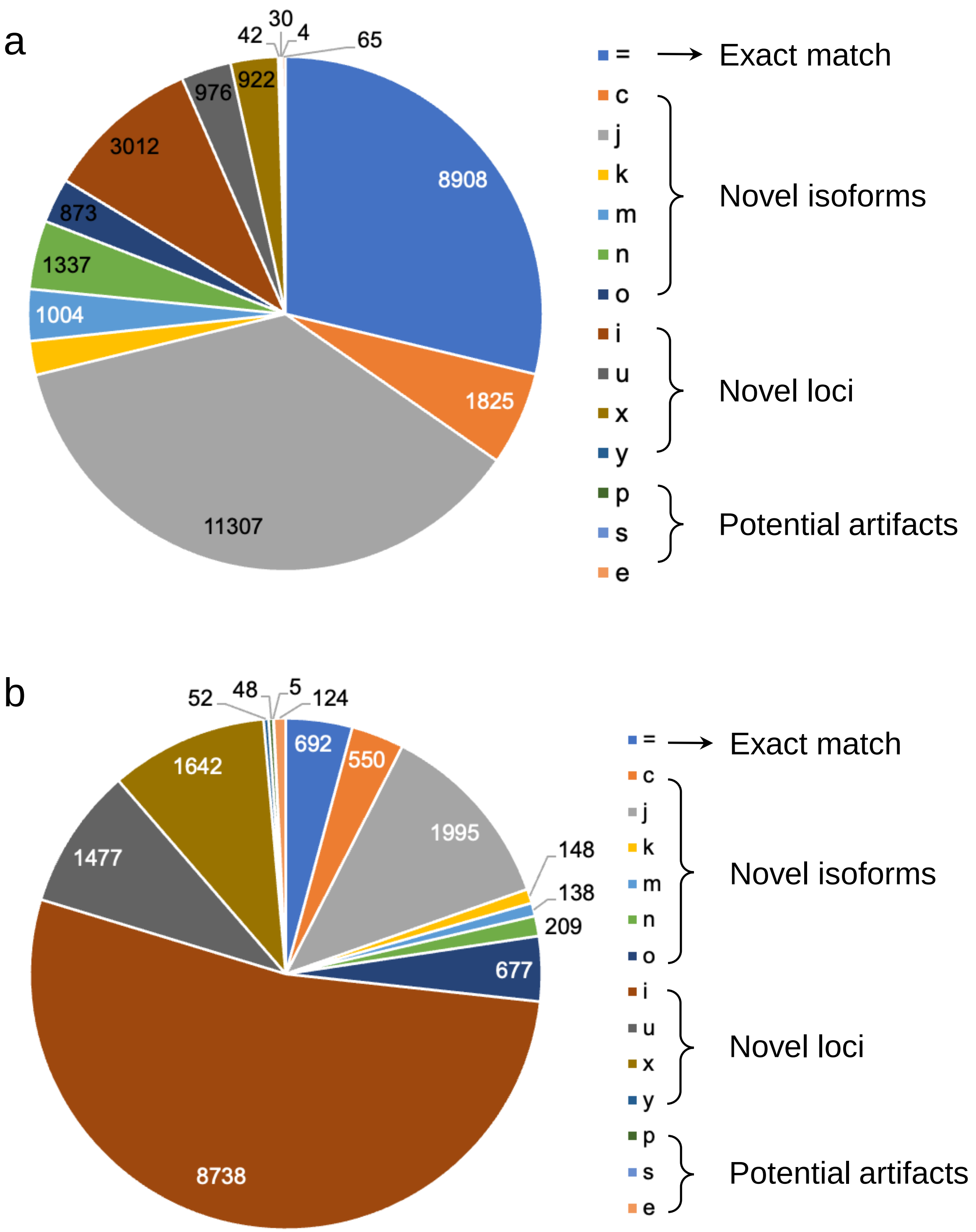

Supplementary Figure 4. GffCompare types when comparing protein-coding (a) and lncRNA loci (b) predicted in this study with those predicted in Jehl et al. (2020). Transcripts were classified into: 1) Exact match: GffCompare code "=", which means the intron chains of our annotated transcripts can exactly match to reference annotations; 2) Novel isoform: GffCompare codes 'c,' 'k,' 'j,' 'm,' 'n,' or 'o', which means predicted transcript cannot match a reference transcript but can match a reference gene; 3) Novel loci: GffCompare codes 'i,' 'u,' 'y,' or 'x', which means predicted transcript cannot match either a reference transcript or a reference locus.

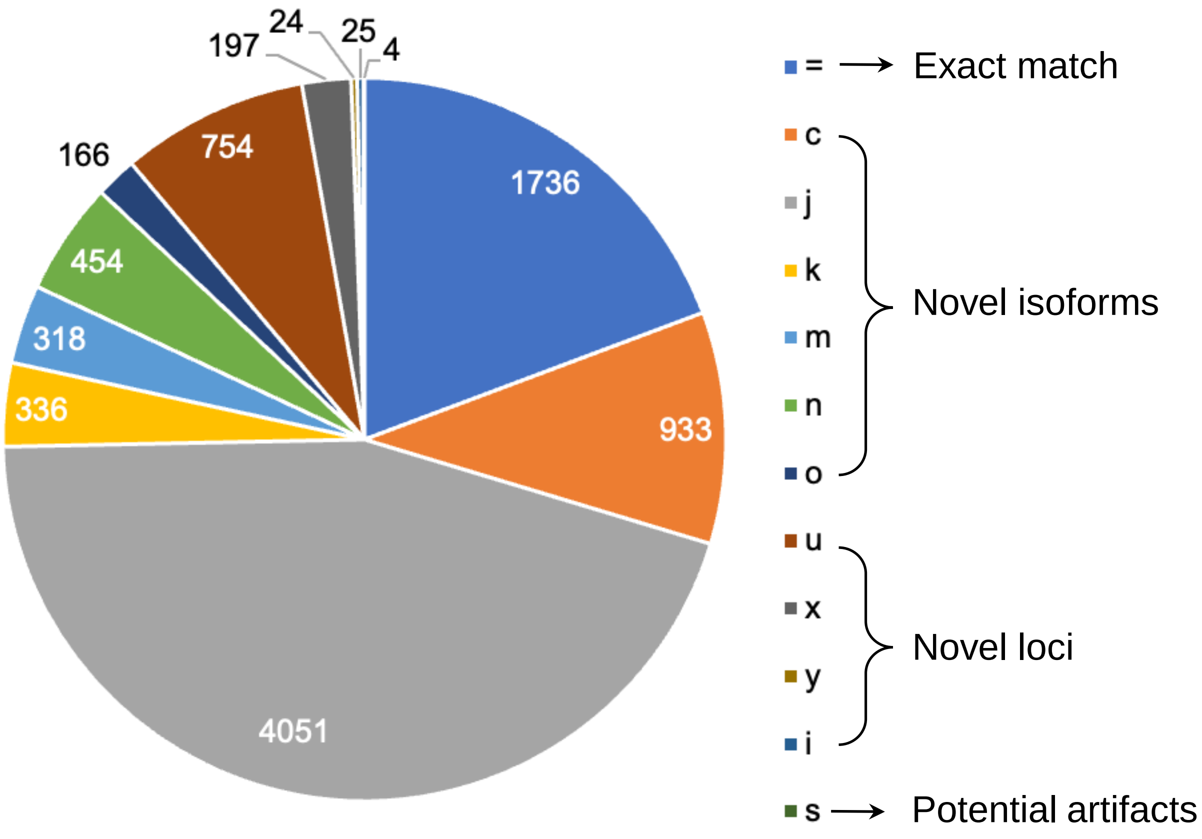

Supplementary Figure 5. GffCompare types when comparing novel transcripts reported by Thomas et al. (2014) to our annotation. Transcripts were classified into: 1) Exact match: GffCompare code “=”, which means the intron chains of our annotated transcripts can exactly match to reference annotations; 2) Novel isoform: GffCompare codes ‘c,’ ‘k,’ ‘j,’ ‘m,’ ‘n,’ or ‘o’, which means predicted transcript cannot match a reference transcript but can match a reference gene; 3) Novel loci: GffCompare codes ‘i,’ ‘u,’ ‘y,’ or ‘x’, which means predicted transcript cannot match either a reference transcript or a reference locus.

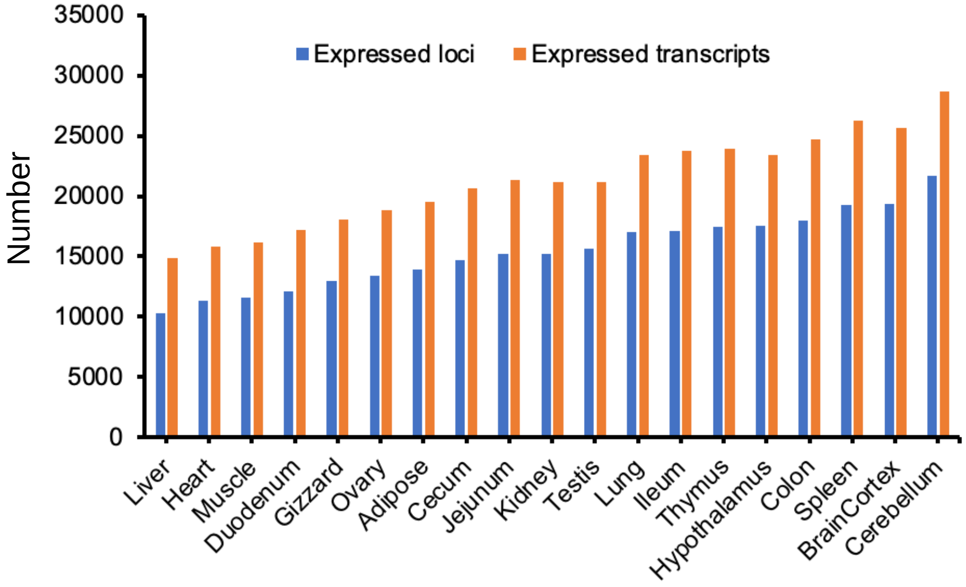

Supplementary Figure 6. Number of expression loci and transcripts (TPM > 0.1) across tissues

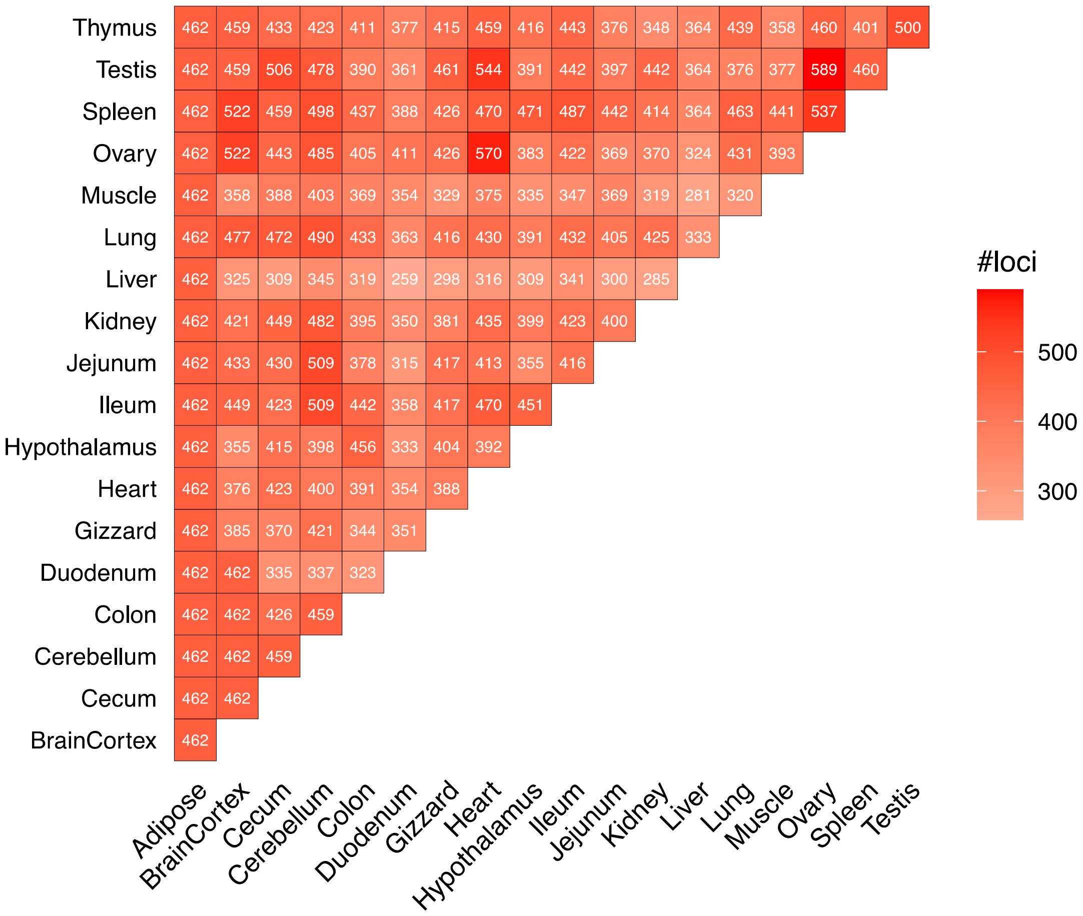

Supplementary Figure 7. Number of loci showing differential alternative splicing between tissues
